# Predicting Firefighters’ Physical Ability Test Scores from Anaerobic Fitness Parameters & Mental Toughness Levels

**DOI:** 10.3390/ijerph192215253

**Published:** 2022-11-18

**Authors:** Peter Beitia, Andreas Stamatis, Tal Amasay, Zacharias Papadakis

**Affiliations:** 1 Human Performance Laboratory, Department of Health Promotion and Clinical Practice, College of Health and Wellness, Barry University, Miami Shores, FL 33161, USA; 2 Exercise and Nutrition Sciences, State University of New York, Plattsburgh, NY 12901, USA

**Keywords:** tactical athlete, load carriage, PPE, SCBA, tactical performance, physical fitness, psychological components, mental health

## Abstract

Physical ability test (PAT) evaluates firefighters’ (FF) occupational capacity. The contribution of anaerobic systems during PAT and mental toughness (MT) relationship to PAT is unexplored. PAT modeling based on anaerobic fitness (AF), MT, and respective relationships were examined. Fourteen male FFs (Age: 29.0 ± 7.0 years) completed a PAT composed of occupationally-specific tasks in full gear. On a separate day, a series of AF assessments were performed (handgrip-dynamometry: HG; vertical-jump: VJ; Margaria-Kalamen: MK; 300-yard shuttle run: 300YD). MT was evaluated using military training MT inventory (MTMTI) and sports MT questionnaire (SMTQ). We tested the PAT model using multiple backward regression and related correlations coefficients at *p* < 0.05. A 78% proportion of PAT was explained by AF parameters (F_2,13_ = 20.2, <0.05). PAT was significantly correlated with HG (r = −0.71, *p* < 0.01), VJ (r = −0.73, *p* < 0.01), MK (r = −0.75, *p* < 0.01), and with 300YD (r = 0.60, *p* < 0.05). MT did not demonstrate significant correlation with PAT (*p* > 0.01). Anaerobic system significantly contributes to PAT performance. FFs should optimize AF training, which would allow for enhanced occupational performance in PAT. Further investigation into psychological determinants of FFs is recommended.

## 1. Introduction

Firefighting is known to be highly stressful, challenging, and full of immense physical and emotional pressures [1,2,3,4,5,6,7]. The physical demands of firefighting depend on the task and on the unpredictable working conditions [2,8,9,10]. Firefighters’ (FF) work must be done with time urgency and is often performed under the psychological stress of knowing that civilians are in imminent danger [9,10,11,12]. Additionally, FFs must perform their work while wearing personal protective equipment (PPE) and self-contained breathing apparatus (SCBA) equipment. This is necessary to protect the FF while they perform. Most of their time, their duties are performed under life-threatening environments. On top of that, such protective gear imposes a considerable physiological burden because of its weight, insulative properties, and restrictiveness [12,13,14,15,16,17]. These unique sets of stressors cause FFs to exhibit extreme/substantial physiological and psychological responses such as fatigue, over-exhaustion, and impaired cognitive function [1,6,11,18,19,20,21]. Such responses present a high occupational hazard and may compromise FFs’ health and safety, including the risk of injury and even on-duty death compared to other occupations [10,18,22].

Increased physical fitness has been shown to enhance job performance and decrease injuries and disabilities in FFs while on-duty [23]. Research over the past several decades has led to the acceptance/application of minimal physical fitness standards, as recommended by various organizations (i.e., National Fire Protection Agency, International Association of Firefighters, and the International Association of Fire Chiefs) [24]. According to these recommendations, the physical ability test (PAT) was developed to evaluate FFs’ occupational performance and readiness [25,26,27,28,29]. There has been considerable attention paid to the assessment of aerobic requirements related to firefighting; however, less focus has been considered for the measurement of anaerobic contributions to occupation-specific metabolic demands [30]. There is a need to specify how anaerobic performance and, more specifically, anaerobic fitness (AF) parameters (i.e., peak anaerobic power, anaerobic power, anaerobic capacity, and fatigue index) are equal or less important to already established aerobic fitness parameters of firefighting.

Given the anaerobic nature of many firefighting tasks and the sizable cohorts of firefighting personnel, the inclusion of field assessments that provide valid and reliable measurement scores of AF is warranted. For example, the simplistic testing nature of the handgrip (HG) dynamometry, maximal vertical jump (VJ), Margaria-Kalamen (MK) staircase test, and 300-yard (274 m) shuttle run, individually may capture a sole dimension of anaerobic performance (i.e., peak anaerobic power, anaerobic power, anaerobic capacity, and fatigue index) [24,31]. However, when all are implemented in a battery format, they may capture a complete understanding of anaerobic performance that is occupationally specific, which allows for cohort testing applicability, and which is time-efficient and economically feasible amongst FF populations.

However, according to Michaelides et al. [32], “fitness parameters used alone in prediction models are not sufficient to fully predict a FF’s job performance.” Due to the high occupational demands placed upon FFs, psychological skills may facilitate a FF’s ability to cope with the adversities of the occupation, as well as to promote optimal performance in arduous and intense firefighting tasks [33,34,35]. More recently, there has been a concomitant emphasis and resurgence of interest in enhancing both physical and mental toughness (MT) by integrating mental and physical strength and conditioning principles [28,29,32,33,35]. Yet, there have been no recordings of MT and changes in FFs’ MT in response to physiological strains, nor has MT’s influence on recruitment or incumbent FFs’ occupational performance in the PAT been recorded. In addition, there is no MT measurement instrument specifically designed for FF populations [7,33,36,37]. Therefore, due to the aforementioned lack in the MT related literature, incorporating the military training mental toughness inventory (MTMTI) [36] and sports mental toughness questionnaire (SMTQ) [38] as MT psychological assessments, may allow one to better predict FFs’ performance on specific firefighting tasks, including the PAT.

To the best of our knowledge, there is no research available investigating the influence of both physiological and psychological parameters (i.e., MT) to predict FFs’ PAT performance. An interdisciplinary assessment that encapsulates the complete physiological and psychological demands of firefighting, pertaining to AF parameters and a MT psychological component, may provide a holistic understanding of the firefighting occupation and help FFs improve performance on specific firefighting tasks and the PAT. Such an integrative approach, when creating a prediction model for FF performance as evaluated by the PAT, may unfold more of the unexplained variance in prior research [32,39,40].

Therefore, the purpose of this research study was: (a) to create a model to best predict recruit or incumbent FFs’ performance time in the PAT scores based on AF parameters and/or MT psychological component; and (b) to identify the relationship between AF parameters and the MT psychological component with firefighting performance, as evaluated by the PAT.

## 2. Materials and Methods

### 2.1. Experimental Design

A repeated cross-sectional quantitative research design was employed to investigate the correlation between the independent variables (i.e., HG dynamometry, maximal VJ, MK staircase test, 300-yard shuttle run, MTMTI instrument, and SMTQ instrument) with the dependent variable of PAT performance time score, which is the industry standard for ensuring the minimum fitness levels amongst active fire suppression personnel [27].

This research study consisted of recruited cohorts from two local fire departments, all who volunteered to participate and performed their respective department’s PAT. Due to various reasons, including shift changes, vacation leaves, medical leaves, job-related injuries, and the global COVID-19 pandemic, only 14 male FFs completed all the measurements.

Experimental conditions for data acquisition were completed and obtained on two different days separated by, at minimum, 48 h and, at maximum, one week apart. This was done in order to effectively provide information about FFs’ firefighting ability and physical conditioning, as well as to minimize fatigue induced by the respective physical performance bouts. On the first day, participating FFs performed a PAT at their respective fire department facility center from the South Florida area. Then, with no more than seven days apart, participants returned to the same facility center or the Barry University at Human Performance Laboratory (HPL), to perform a series of physiological AF assessments, including HG dynamometry, maximal VJ, the MK staircase test, and the 300-yard shuttle run.

FFs initially presented themselves with shorts, t-shirts, and a pair of comfortable jogging/running shoes. At first, anthropometric and body composition analyses were performed, followed by the respective experimental condition in full occupational firefighting gear, including PPE (i.e., firefighting pants, jacket, boots, bella cava, helmet, gloves, Scott harness, and pack) and SCBA. FFs used the SCBA during their respective department’s PAT. However, they did not have to breathe through their SCBA for the AF assessment condition. The MT psychological component in FFs was assessed once after the completion of the PAT testing and once after the completion of the AF assessments via a MTMTI and SMTQ instrument.

### 2.2. Subjects

Volunteers were eligible to participate in the research study if they met the following characteristics: male recruits or incumbent active fire suppression FFs from respective fire departments in the South Florida area (i.e., City of Coral Gables Fire Department [CGFD] and Hialeah Fire Department [HFD]), between the ages of 20–55 years, apparently healthy and cleared to perform their occupational duties by their respective fire department. Participants were informed of the experimental risks and signed an informed consent document before the investigation. The investigation was approved by the Barry University Institutional Review Board for use of human subjects.

### 2.3. Procedure

Prior to data collection, height (cm) and weight (kg) were measured using a stadiometer and SECA weight scale (Model #7802321138), and BMI as mass (kg)/height (m^2^) was calculated. Next, in accordance with guidelines set forth by the American College of Sports Medicine (ACSM), we obtained circumferential measurements at the hip and the waist [41]. Waist and hip circumferences were measured twice and recorded to the nearest 0.1 cm. Waist to hip ratio was calculated by dividing the waist by the hip girth [41]. The body composition analysis was conducted using a bioelectrical impedance analysis, Omron Body Fat Analyzer (Model #HBF-306BL), obtaining the FF’s body fat percentage. FFs were instructed to adhere to the following guidelines before anthropometry and body composition testing: (a) no urination and/or void within 30 min of the test, (b) no eating or drink within 4 h of the test and no use of diuretic agents (e.g., caffeine and chocolate) within 12 h of the test, (c) no alcohol consumption within 12 h of the test, (d) and no exercise within 12 h of the test. All guidelines were repeated for both the experimental conditions [41]. Each FF was asked to stand upright, contacting the electrodes of the analyzer. Procedures for this test were conducted following the equipment manual [41].

#### 2.3.1. Experimental Condition 1—PAT

The CGFD and HFD use PATs that are similar to the national candidate-PAT (CPAT) for their annual PAT screening/maintenance occupational performance and readiness program [26]. However, unlike in the CPAT, which requires FFs to wear a 22.68 kg (50 lbs.) vest to simulate the weight of SCBA and PPE, both CGFD and HFD PATs were done in full occupational FF gear, including PPE and usage of SCBA (~23 kg). All FFs needed to have a standardized PPE, which is provided by their respective departments, with the option to add more or substitute some equipment from vetted vendors and from a standardized list of approved equipment. FFs have the option to keep their PPE either at home or at the station, but the SCBA is always kept at the firehouse at which they are located. Both department PATs are in conjunction with Fire Service Joint Labor Management Wellness/Fitness Initiative of the IAFF and the IAFC. Before performing the PAT, FFs executed their respective standard department warm-up. All PATs were administered to all participants by respective fire department trained instructors, and our research team did not have any involvement in the administration of the PAT. No testing was performed prior to the PAT or on the day prior to performing the PAT. The events were done sequentially, and the clock did not stop until all of part one tasks were completed. Tasks were separated by distances of 29.08 m (85 ft). FFs were advised to pace themselves and were required to walk during these intervals (i.e., no running was allowed at any time during the test). The sum of each task and transition constitutes the cumulative time, measured in seconds. A passing score was a cumulative performance time less than or equal to ten minutes twenty seconds, as well as the ability to complete all the tasks with satisfactory performance (i.e., pass). Below are brief description of the CGFD and HFD PAT. Further description of the respective PAT available within the supplementary material.

CGFD PAT

Similar to the CPAT, the CGFD PAT was comprised of eight complete tasks. FFs were timed from the initial start and for the duration of each task, as well as during each transition using standardized procedures for all eight tasks, as described by CGFD. The CGFD PAT tasks included a stair climb, hose drag, equipment carry, ladder raise and extension, forcible entry, search, rescue, and ceiling breach and pull. A passing score was a cumulative performance time less than or equal to ten minutes twenty seconds, as well as the ability to complete all the tasks with satisfactory performance (i.e., pass). The PAT performance time score served as the dependent variable in the current research study [26].

HFD PAT

The HFD PAT was comprised of two parts with nine complete tasks. FFs were timed from the initial start and for the duration of each task, as well as during each transition using standardized procedures for all nine tasks, as described by HFD. The first part consisted of five tasks which were completed sequentially within seven minutes and tested the FFs’ physical fitness ability. The second part consisted of four separate tasks, testing a FF’s ability to perform occupational tasks as a FF; part two had no time limit. However, performance is based on a pass or fail criterion. The HFD PAT tasks included a stair climb, hoist evolution, forcible entry, hose advance, a victim (mannequin) drag rescue, ladder carry, ladder climb, extrication exercise, and confined space crawl. A passing score was a cumulative performance time score less than or equal to seven minutes, as well as the ability to complete all of part two tasks with satisfactory performance (i.e., pass). The first part of the PAT (i.e., performance time score) served as the dependent variable in the current research study [42].

#### 2.3.2. Experimental Condition 2—Anaerobic Fitness (AF) Assessments

FFs performed a series of physiological AF assessments, including HG dynamometry, maximal VJ, the MK staircase test, and the 300-yard shuttle run. For the current research study, the AF assessment’s general elapsed testing duration determined the experimental testing protocol sequence (i.e., shortest to longest). Each individual assessment protocol was separated by a rest period of five minutes until completion of the test series to prevent the effects of fatigue from confounding test results, allowing for optimal performance/injury prevention within each individual assessment protocol and complete restoration of the anaerobic energy system. Participants were continually monitored during each individual assessment protocol and allowed to stop at any time. Every effort was made to minimize all the physiological risks inherent with vigorous exercise through preliminary screening, identification of contraindications to exercise testing, adherence to standards of practice for AF assessment protocols, and personal monitoring of each assessment by the principal investigator and research team. All events were monitored and recorded by a certified strength and conditioning specialist (CSCS)/tactical strength and conditioning facilitator (TSAC-F). Individual assessment methodologies for HG dynamometry, maximal VJ, MK staircase test, and 300-yard shuttle run are described below [43].

Handgrip (HG) Dynamometry

Upper-body strength was assessed using a HG dynamometer (Model #T.K.K. 5001; Takei Scientific Instruments Co., Ltd., Tokyo, Japan). The procedure for the HG dynamometry was administered following the instructions from Beam and Adams [44]. The grip size of the force dynamometer was set at maximum grip size (7) to allow the FF to fully grasp the dynamometer while wearing their tactical gloves. The best of three trials on each hand was registered as the maximal pressure in kilogram (kg). The highest measurement for each hand was recorded, and the sum of the high scores for each hand was used for analysis.

Maximal Vertical Jump (VJ)

The maximal VJ test was conducted using the Vertec^®^ Jump Measurement System. The procedure for the VJ test was administered following the instructions from Beam and Adams [44]. The FF completed at least three maximal trials (or continued until the jumping reach height plateaus), with 60 s rest between trials. The technician recorded each trial, or simply the highest jumping reach height (to the closest 0.5 in), and was later converted to metric units. Peak power (PP) recorded in watts (W) was calculated using the Sayers equation, as follows: PP = [51.9 × Counter Movement Jump height (CMJ; cm)] + [48.9 × Occupational Weight (kg)] [45].

Margaria-Kalamen (MK) Staircase Test

The MK staircase test was designed to test anaerobic power; specifically, because of its relatively short duration (usually less than five seconds). It tests the contribution of the phosphagen system [46]. The procedure for the MK staircase test was administered on a staircase following the instructions from Haff and Dumke [47]. The principal investigator instructed the FF to run up the stairs as quickly as possible; the timer started when the FF stepped on the third step and stopped when the FF stepped on the ninth step. The test was repeated three minutes after completing the first trial. The mean time was calculated based on trial one and trial two.

300-Yard (274 m) Shuttle Run

Implementation of the 300-yard shuttle run test, first described in by Gilliam and Marks [48], was used to measure anaerobic power, anaerobic capacity, and fatigue index (FI) resistance through its repeated maximal sprint bout protocol [49,50,51]. The 300-yard (274 m) shuttle run test, through intermittent 25 yds (22.86 m) accelerations interspersed with a recovery period, served to evaluate participants’ AF as it relates to FF performance. The mean time value of the two performed bouts constituted the result of the 300-yard shuttle run test. The procedure for the 300-yard shuttle run test was administered following the instructions from Gilliam and Marks [48].

Psychological Inventories—Military Training Mental Toughness Inventory (MTMTI) and Sports Mental Toughness Questionnaire (SMTQ)

The MTMTI was used to measure MT from a tactical athlete population behavioral perspective [36]. The MTMTI is a third-party reported six-item instrument established to ascertain military personnel’s MT levels. A commanding FF officer and a second assessor (i.e., either another supervisor or a fellow FF personnel) from the respective fire department were asked to complete the instrument on behalf the FF for the research study. Two distinct assessors were used to improve interrater reliability and not have a biased perception with how other peers/supervisors perceive FFs’ MT. The assessors were asked to rate how well the FF was able to maintain a high level of personal performance when confronted with different stressful situations in their occupation (for example—items include “when the conditions are difficult” and “when he has been reprimanded or punished”). Responses were based on a seven-point Likert scale that ranges from 1 (never) to 7 (always), with a midpoint anchor of 4 (sometimes). Total MTMTI scores are a composite of the scale’s six factors (i.e., confidence, constancy, control, resilience, and confidence).

FFs participating in the research study were asked to complete the SMTQ [38]. The SMTQ is a self-reported 14-item instrument established to ascertain athletes’ MT levels. The FFs responded to items on a four-point Likert-type scale ranging from “not at all true” to “very true.” Sample items included “I interpret threats as positive opportunities” (confidence); “I give up in difficult situations” (constancy); and “I am overcome by self-doubt” (control). Total SMTQ scores are a composite of the scale’s three factors (i.e., confidence, constancy, and control).

To encourage honest responding, FFs were informed that the index/questionnaire is for research purposes only and that their responses were kept confidential and would not influence their occupational duties. No time limit was imposed on the FFs for filling-in the inventory/questionnaire. FFs were evaluated by a commanding firefighting officer and a second assessor (i.e., either another supervisor or a fellow FF) via the MTMTI once after the PAT and once after the battery of AF assessments; this, in part, allowed for improved inter-rater reliability and consistency regarding how others perceive the assessed FF’s MT. Upon evaluation of MT via MTMTI, the assessors immediately handed over the instrument recording form to a member of the research team for data retention and anonymity/confidentiality purposes. FFs were asked to take the self-reported SMTQ once after the PAT and once again after the battery of AF assessments; this, in part, allowed for improved intra-rater reliability and consistency regarding how FFs perceive their own MT. FFs completed the instrument and returned it back to a member of the research team for data retention and anonymity/confidentiality purposes. FFs’ MT was assessed after the PAT and after the battery of AF assessments to observe the relationship of MT and their performance after undergoing an arduous and intense physiological bout. This allowed the research team to observe FFs’ ability to cope with the adversities of the occupation. The purpose of collecting data twice from the instruments was to promote statistical analysis in future studies (i.e., observe for any differences in MT between performance PAT and performance in different physical battery format testing, assess the concurrent validity of the MTMTI and SMTQ amongst FFs, and other tactical athlete populations, as well as potential applications in longitudinal studies of similar cohorts). Since the demands of firefighting depend on the task and on the unpredictable working conditions that pose heavy physical and mental stress on FFs [1,2,5,6,10,11,18,20,52,53,54], our research team observed how MT is influenced by the tasks they are asked perform.

### 2.4. Statistical Analyses

A convenience sample was used based on the class’s size from two different FFDs. Therefore, a sample size calculation was not performed based on the available literature and the examined variables [55]. Both FF null bodyweight (i.e., bodyweight in shorts and t-shirt only), as well as full occupational FF weight (kg), including all PPE and SCBA gear, was used for statistical analyses. However, for the experimental protocol’s AF assessments, the power values used only took into consideration the FFs’ occupational weight. This was done in order to account for potential discrepancies due to the physiological strain imposed by the extreme tactical load carriage. Absolute power measurements were calculated using full occupational FF weight as reference.

Means and standard deviations were calculated for all variables. Data were examined for normality and proper adjustments were made in case of any violations of additivity/linearity, normality, multicollinearity, homoscedasticity/homogeneity of variance, and independence. Pearson-product moment correlation coefficients were calculated among the physiological and psychological variable scores and the PAT performance time scores in an attempt to identify the importance of each parameter with relation to firefighting occupation performance in PAT. Multiple regression analysis was used to determine the proportion of the variation observed in the PAT performance (dependent variable) that is explained by the variation of the important physiological and/or psychological parameters (independent variables). Therefore, based on the independent variables used, we could best predict the FF’s performance time scores in the PAT. The backward selection method was initially used in an attempt to identify the subset of useful independent variables (i.e., HG dynamometry represented in absolute strength (kg), maximal VJ represented in absolute peak power (W), MK staircase test represented in absolute power (W), 300-yard shuttle run represented in mean trial time score (s), MTMTI instrument represented in mean score of both experimental conditions, and SMTQ instrument represented in mean score of both experimental conditions) and correct for any potential multicollinearity [56,57]. All statistical analyses were done using IBM SPSS 27.0 (SPSS, Inc., Chicago, IL, USA) and significance accepted at *p* < 0.05, unless otherwise stated.

## 3. Results

The recruited cohorts from CGFD (*N* = 9) and HFD (*N* = 5) completed their respective department’s PAT. Detailed descriptive statistics for the participants are presented in Table 1. The PAT and AF assessment sessions took place throughout the year of 2020 and 2021. FFs’ performance on the PAT and various AF parameters are displayed in Table 2. FFs’ MT levels, as assessed via the two MT psychological component instruments, are displayed in Table 3. The environmental conditions were recorded with a national weather station monitor (Miami; KHB34; 162.550; Miami, FL, USA). The average temperature and humidity during the sessions were 28.8 degrees Celsius and 73%, respectively.

### 3.1. Correlations between PAT Scores, AF Parameters and MT Levels

Pearson-product moment correlation coefficients were calculated and used to identify the relationships among the AF parameters and MT levels assessed via the two MT psychological component instruments, with the firefighting performance as evaluated by PAT performance time scores as shown in Table 4.

### 3.2. AF Parameters and/or MT Levels to Predict PAT Scores

Multiple backward regression analysis was used to predict PAT performance time scores using AF parameters and/or MT levels assessed via the MT psychological component instruments. A model including only anaerobic physiological fitness parameters better predicted a FF’s performance in the PAT, as illustrated by Table 5. As noted, prior MT levels did not demonstrate any significant correlation with PAT performance time scores. Therefore, using a prediction model that included both AF parameters and MT levels assessed via the two MT psychological component instruments to better predict FFs’ performance in the PAT was not concluded. A significant proportion (78%) of the variation observed in the PAT scores was explained by the variation of the AF parameters, specifically with the 300-yard shuttle run test and the maximal VJ, as illustrated by Table 6. Further statistical results including Table S1. ANOVA–PAT Scores, AF Parameters and MT Levels available within the supplementary materials.

PAT Performance Time Score = 30.1 − [(0.06) × (Vertical jump, absolute peak power in watts)] + [(6.89) × (300-yard (274 m) shuttle run test, mean time score of two trials in minutes:seconds)](1)

## 4. Discussion

The purpose of this research study was (a) to create a model to best predict the FF performance time in the PAT scores based on AF parameters and/or MT levels assessed via the two MT psychological component instruments, and (b) to identify the relationship between physiological and psychological parameters with firefighting performance, as evaluated by the PAT scores. AF assessments and MT psychological component instruments were hypothesized to correlate with FFs’ performance time score in the PAT.

HG, VJAPP, and MKAP all demonstrated significant negative correlation, meaning that the better a FF’s performance at each individual assessment, the more of a decrease in PAT performance time score (seconds). The 300-yard (274 m) shuttle run test revealed a significant positive correlation, demonstrating that the longer a FF took to perform the 300YD, the more of an increase in PAT performance time score. Amongst military personnel, the MTMTI and SMTQ demonstrated the ability to predict performance, however, the MT psychological component instruments did not demonstrate a relationship with FFs’ PAT performance.

### 4.1. Anaerobic Fitness and Physical Ability Test

Our research findings confirmed that the tasks presented in the PAT, such as forcible entry or ladder retrieval, require a sustained high force and metabolic output and, logically, all aspects of anaerobic performance and specifically AF parameters (i.e., anaerobic power, peak anaerobic power, anaerobic capacity, and fatigue index) would be related to optimal performance in the PAT. Williams-Bell et al. [40] confirmed such a notion when their research found respiratory-exchanges ratios in excess of 1.0 during a simulated ability test on fifty-seven (23 being women) candidate FFs, implying significant activation of anaerobic metabolism. There have also been numerous reports of firefighting tasks having strong anaerobic component ranging from 35 to 60% of the metabolic demands during structural firefighting activity [23,24,52,58]. More specifically, Bilzon et al. [58] found that amongst 49 Royal Navy FFs (34 males, 15 females), performing several 4-min simulated shipboard firefighting tasks, required 35% to 41% of the energy demands to be met anaerobically, while Davis and Dotson [23] reported anaerobic contributions equivalent to approximately 40% [23,52]. During various firefighting activities, FFs were required to work at up to 97% VO_2max_ [23], and blood lactate values were reported to be as high as 13 mmol L^−1^ [2]. Heimburg et al. [4] characterized the physiological responses of 14 FFs during a training course, which simulated a hospital rescue correlating baseline fitness parameters with total performance time; the fast performers were shown to have a significantly lower accumulated oxygen uptake (16.9 ± 1.5 mL·kg^−1^ vs. 19.9 ± 1.4 mL·kg^−1^) compared to the slower performers, respectively, implying a lower reliance on anaerobic metabolism. Similarly, Bilzon et al. [58] found that FFs with a maximum oxygen consumption (VO_2max_) greater than 43 mL·kg^−1^·min^−1^ were able to work more aerobically, and participants with a lower VO_2max_ were able to complete the same amount of work. However, they did so by working at a higher percentage of VO_2max_ and producing more energy anaerobically. Regardless of differences in research methodologies, altogether the research findings suggest that FFs with greater AF may be able to continue to function at a high level during simulated (i.e., PAT) or actual firefighting emergency scenarios, less anaerobically fit individuals may be required to cease current tasks to recover from prior maximal and anaerobic exertion. The intermittent nature of firefighting and the reliance on anaerobic metabolism support the inclusion of AF assessments amongst FFs and support the results of our research study, which highlights specific AF parameters, such as anaerobic power, peak anaerobic power, anaerobic capacity, and fatigue index.

### 4.2. Handgrip Dynamometer and Physical Ability Test

Hauschild et al. [25], in a systematic review of correlations between fitness tests and discrete occupational tasks amongst 27 different studies within the tactical athlete population (13 military population; 10 FF, law enforcement, or peace officer; and 4 healthy civilian populations), found that HG dynamometry test was strongly correlated (*r ≥* 0.5) with one-quarter (i.e., lift and lower [single] task with *r* = 0.67, *p <* 0.05; lift and lower [repeated] task with *r* = 0.59, *p <* 0.05; stretcher carry task with *r* = 0.61, *p <* 0.05) of the 12 occupational tasks categories by which the authors examined relationships with it. However, the mean maximal HG strength in studies of FFs’ work capacity range from 47 to 61 kg for men, showing that the maximal HG strength in our current research study (33.1 kg ± 7.8 kg) does not fall within norms of previous research studies [32,40,52,59,60,61,62,63,64,65,66,67,68,69,70]. Such discrepancy may be explained by the fact that, in our protocol, FFs wore their full occupational firefighting gear including their tactical gloves, while the prior research studies either did not use PPE, used parts of it, or did not use a control trial. Moreover, reasons to explain the aforementioned inconsistency of our results with previous studies may be because of the PPE, which has been characterized to have a negative impact on FF performance [17]. Lesniak et al. [15] found PPE to cause movement restriction amongst FFs when quantifying the detrimental effect of load carriage (including PPE and SCBA) on FF occupational performance in a simulated fire ground test. The movement restrictions coupled with test–retest reliability on the HG, which can be affected by several factors, i.e., calibration, use of one or both hands, number of attempts and pre-post maximal exertion, which may explain the difference in HG performance range within our research study as compared to other studies. The PPE-induced movement restriction may have inhibited our FFs’ ability to fully grasp object(s) because of the gloves’ clothing restriction. Regardless of prior limitations, HG dynamometry provides another standard measurement for accessing the muscular strength of the upper body, as it requires maximal muscle actions that generally are based upon rapid onset and decay of peak force where the apparent energy pathway is predominantly the phosphagen system and, therefore, is an accurate means of measuring AF parameters [44]. Williford et al. [69] found that amongst 91 FFs, total HG strength proved to be the best association of total task time (*r* = −0.54, *p* < 0.01) in a physical performance assessment (similar to PAT), with higher levels of strength predicting lower task time in the simulated job performance test. Davis et al. [60], who compared baseline fitness and anthropometric characteristics with performance in firefighting tasks, found that HG strength, along with other variables, were predictors of performance (*R*² = 0.63, *N* = 100). More recent studies conducted by Rhea et al. [66] and Sell [68] demonstrated that FFs’ HG strength was found to be above the 95th percentile when compared to age-sex corrected norms. Both the Sell [68] and Rhea et al. [66] studies used relatively small samples (*N* = 21 and *N* = 20, respectively), similar to this current research study, although Sell’s sample size was that of ‘hotshot’ FFs, a highly trained and specialized firefighting force [68]. Additionally, Rhea et al. [66] demonstrated that HG strength was significantly correlated with total task performance and had correlations identical to our current research study (*r* = −0.71, *p* < 0.05). Despite the differences in methodology, HG absolute strength measured via a dynamometer appears to be a significant correlator of FF occupational tasks and, in the case of this research study, a significant correlator with PAT performance.

### 4.3. Vertical Jump and Physical Ability Test

Hauschild et al. [25], who examined the relationship between fitness assessments and discrete occupational task found the jump test (e.g., VJ) to be strongly correlated (*r ≥* 0.5) with one-third (i.e., lift and lower (single) task with *r* = 0.52, *p <* 0.05; move fast task with *r* = 0.60, *p <* 0.05; multi-activity task with *r* = 0.52, *p <* 0.05) of the 12 occupational task categories. VJ values have been classified as average amongst Sell’s [68] ‘hotshot’ FFs compared to sex- and age-corrected norms. Michaelides et al. [32] found that relative anaerobic power in the VJ was a moderate, statistically significant correlator with total time to perform the PAT, as well as significantly correlated with individual firefighting tasks, charged hose advance (*r* = 0.28, *p* < 0.05), and rescue mannequin drag tasks (*r* = 0.31, *p* < 0.05). Similarly, our research study noted maximal VJ represented in absolute peak power was statistically significant and negatively correlated (*r* = −0.73, *p* < 0.05) with PAT performance. Contrary to Michaelides et al. [32], our research study did not observe for correlations between fitness assessments and discrete firefighting tasks. Additionally, unlike Michaelides et al. [32], our research study used absolute values for power measurement, which still incorporates full occupational firefighting weight into the data output rather than reducing maximal workload output by dividing watts per kilogram of full occupational FF weight and inducing relativity and normalization of power measurement. The notion that it may not be appropriate to normalize testing results to body mass was furthered by Fyock-Martin et al. [30], who stated that absolute-based power measurements may be more occupationally specific and indicative of performance because, during PATs, FFs are required to move their own relative bodyweight. However, PATs are conducted in standard firefighting equipment (absolute), using protective gear with a fixed weight (absolute), and with the potential involvement of civilian bodyweight (absolute). All the occupational demands suggest it may be best to assess AF parameters in absolute terms, as opposed to relative terms.

### 4.4. Stair Climbing and Physical Ability Test

Stair climbing protocols have been used amongst researchers to assess anaerobic performance in various tactical athlete populations, including FFs [24,32,61,71,72,73]. Clarke [24] introduced a tower climb test (TCT) to assess anaerobic performance in urban FFs and found that staircase protocols provided an occupationally specific mean by which to measure anaerobic performance. Michaelides et al. [32] found that, amongst 90 FF participants, the relative anaerobic power (normalized to body mass/no PPE) in the 1 min anaerobic step test (AST) was significantly and negatively correlated with total time to perform the PAT (*r* = −0.40, *p* < 0.05). Houck et al. [61] are the only researchers, to our knowledge, having incorporated the MK staircase test, a valid and reliable standardized staircase protocol amongst the tactical athlete populations, specifically on FFs. Their research study examined the physical fitness of 77 wildland FFs from the New Mexico Fire Department and compared their results to ACSM normative data and suggested standards for their profession. Their results revealed that 18.75% of participants were ‘‘excellent,’’ 17.5% were ‘‘good,’’ 26.25% were ‘‘average,’’ 25% were ‘‘fair,’’ and 8.75% were ‘‘poor” [61,74]. Our research study’s sample size revealed a mean group data for the Margaria-Kalamen staircase test of 794.5 watts (W) ± 232.7 W (mean score ± standard deviation) with a minimum of 344.0 W and a maximum of 1281.2 W recorded. Our cohort’s recorded power outputs were less compared to Houck et al., whose group mean data were greater and demonstrated power outputs of 1238.5 W ± 326.8 W. However, the physiological testing results of Houck et al. were obtained without incorporation of full occupational firefighting gear, which negates the physiological effects that tactical loading imposes on firefighting performance. Our research study reported MK staircase test in absolute power and was performed in full occupational firefighting gear versus Houck et al. [61] and Michaelides et al. [32], who normalized the data-relative body mass. Calculations of MK staircase test power outputs are mass-specific, so consideration should be made in the interpretation of data. Therefore, regarding anaerobic performance, specifically anaerobic power, during a stair climb, a heavier person generates greater anaerobic stress than a lighter counterpart, even if they score the same time and have covered the same distance [75]. Trends have demonstrated that poorer performance on all the field physical fitness assessments with greater body mass (i.e., greater occupational FF weight) can be attributed to two factors. First, smaller (i.e., lighter) people tend to have a greater strength-to-body-mass ratio based on physiological considerations compared to heavier people whom have been shown to produce lower scores on military physical fitness tests, independent of body fatness [76]. Second, a heavier person tends to be fatter, and the fat weight that must be lifted and accelerated during each jump, running stride, or calisthenic repetition, which detracts from performance [77]. Specifically, within the FF population, the heavy load carriage, along with the PPE and SCBA weight, has been shown to negatively affect physiological responses to work and exercise in laboratory settings [14,16,78,79,80]. The excessive load carriage weight in combination with a MK staircase test protocol has been criticized previously for its 3-step stride pattern and may be challenging for some participants [72], especially because tactical gear is cumbersome and movement-restrictive. Yet, despite these limitations, task familiarity of stair climbing has been previously reported as one of the major benefits of staircase testing [46,81] and may also explain why the MK staircase test represented in absolute power was significantly and negatively correlated to PAT performance (*r* = −0.75, *p <* 0.05). Research studies such as ours need to account for how full occupational FF gear imposes considerable physiological burden due to its weight, insulative properties, and restrictiveness.

### 4.5. 300-yard Shuttle Run and Physical Ability Test

Rhea et al. [66], who looked to identify the relationship between fitness components and job performance within 20 FFs, used a 400-m run for evaluating anaerobic capacity and demonstrated a strong correlation (*r* = 0.79, *p* < 0.05) between total time to complete their simulated test and the AF assessment. To our knowledge, no further research besides ours has been found implementing a sprinting protocol in relation to firefighting performance. Similar to Rhea et al. [66], our research study revealed that the 300-yard shuttle run demonstrated a significant positive correlation (*r* = 0.60, *p <* 0.05) with PAT performance. In spite of differences within sprint distance, our 300-yard (274 m) shuttle run protocol and Rhea et al. [66] 400-m run protocol were applied on similar small sample sizes (*N* = 14 and *N* = 20, respectively), both set out to measure anaerobic capacity (examining the ability of a FF to perform highly intense exertion for medium time spans approximately ranging between 40 to 90 s [48,49,66]), and both used an absolute measure of AF. Potential correlation differences may have been due occupational specificity application, or lack thereof, regarding methodology and test selection. Rhea et al. [66] did not incorporate usage of full occupational FF gear within the fitness assessments. Rhea et al. [66] also did not take into consideration the occupational demands of firefighting when selecting fitness assessment. Firefighting environments are complex, unpredictable, and the fluid nature of the physical environment within a structural fire does not lend itself to linear sprinting, especially at the length of 400 m. The likelihood that FFs would be afforded the space to achieve a sustained straight-line sprint is low. A 400 m run sprint protocol that negates change of direction is not an occupationally specific test selection for FF populations. FFs may need to change tactics, change inertia, and change direction mid-task, most drastically in life or death/escape situations. The 300-yard shuttle run allows demonstration of cognitive coordination and anaerobic power to elicit such changes in direction while performing six round trips of intermittent 25 yds (22.86 m) accelerations as fast as possible without stopping (6 × 50 yd = 300 yds, or 274 m) [48,49]. Two performance bouts are interspersed with a recovery period, putting not only high demands on the anaerobic metabolism during sprints, but also on the aerobic system during rest periods to restore the homeostasis of the intramuscular environment. The 300-yard shuttle run protocol, therefore, assesses FFs’ ability to replicate, at maximum performance, phosphagen system capacity, restoration capabilities, and, specifically, an indicator of anaerobic power, anaerobic capacity, and fatigue index.

### 4.6. Mental Toughness and Physical Ability Test

From a psychological standpoint, MT has been shown to improve the ability to cope [82,83], as well as thrive, under pressure [84]. Since the PAT is a time- and criterion-based assessment stated to objectively measure FF performance and reflect the demands imposed on a FF during actual emergency situations [27], a mentally tough FF may be able to appropriately cope and thrive under such pressure/demands. Therefore, we hypothesized that the MT psychological component would be negatively correlated to the FFs’ performance time score in the PAT (i.e., the more mentally tough, the shorter time to complete the PAT). However, no relationship between MT, as measured with the MTMTI and SMTQ, and FFs’ PAT performance was found.

The relationships between the physical and psychological variables amongst tactical athlete population, specifically military personnel, have been investigated [34,37,85,86,87,88,89,90,91]. Farina et al. [86] assessed predictors of successful selection in the very challenging and stressful United States Army Special Forces Assessment and Selection course among 800 soldiers; several psychological measures were significant predictors, including intelligence quotient, grade level equivalents, resilience score, military aptitude score, and grit (*p* < 0.05). Hammermeister et al. [37] examined the link between “sport” psychological tools and skills with military performers. Their results showed that the mental skills training group had better knowledge and use of mental tools (e.g., goal-setting, self-talk, and relaxation), better knowledge and use of mental skills (e.g., self-confidence, attention control, and the ability to perform more “automatically”), health-related cognitions (e.g., psychological resilience), and outperformed the control group on several physical soldier performance tasks, such as confidence course events and the Army Physical Fitness Test [37]. Research studies regarding exploration of interrelated variables (i.e., physical, and psychological), specifically amongst FFs, are scarce. Gnacinski et al. [33], to date, is the only research study that has explored the relationships between the physical and psychological variables associated with health and performance on candidate FFs. Amongst a sample size nearly double of our current research study (*N* = 34), significant positive correlations (*p* < 0.05) were stated to exist between muscular strength (via 1 RM squat) and conscientiousness (*r =* 0.42) and self-efficacy (*r =* 0.40); muscular strength (via 1 RM bench) and conscientiousness (*r =* 0.40), openness (*r =* 0.37), and self-efficacy (*r =* 0.40); muscular endurance (via push-ups) and conscientiousness (*r =* 0.40), openness (*r =* 0.36), and self- efficacy (*r =* 0.35); as well as between functional movement and extraversion (*r =* 0.42). Research on MT within the tactical athlete population, specifically with FFs, has been predominantly focused on identifying individuals more likely to suffer from stress and stress-related, physical, and psychological illness. Research has revealed high levels of mental stress in FFs associated with exposure to trauma [3] and the emotional demands of work [6]. However, there have been no recordings of MT and changes in FFs’ MT in response to physiological strains, nor has MT’s influence on FFs’ occupational performance in the PAT been recorded.

The lack of MT research and development of MT psychological component instruments for FF population constituted, in the research study, an implementation of two different instruments for measuring MT. The first instrument for MT was the MTMTI, a third-party reported instrument produced by Arthur et al. [36] and used to measure MT from a tactical athlete population behavioral perspective. Similar to military personnel, firefighting action requires FFs to perform under intense pressure in highly stressful environments, characterized by fear, fatigue, and anxiety caused mainly by risk to one’s life. We also evaluated MT from a sports-based approach via the SMTQ, a self-reported instrument [38]. The psychological profile of FFs has shown to be consistent with that of successful athletes furthering the consideration of FFs, categorized as tactical athletes [12,21,33,92]. Elite sports studies share the possible differentiating factors between more- and less-successful performers, including mental imagery, goal setting, positive self-talk, attentional control, etc. Even though we did not measure such differentiating factors, the similarities between athletes and FFs regarding physical and psychological aspects provide a notion to evaluate MT in FFs with the SMTQ and assess its relationship with PAT performance. Both instruments were meant to provide a holistic understanding toward the role of MT in firefighting performance from a tactical athlete standpoint. Usage of both instruments was meant to gain insight into how FFs perceive themselves regarding MT and its relationship to PAT performance, as well as to compare a FF’s MT perception with how other peers/supervisors perceive the FFs’ MT. This, in turn, allowed for compatibility in recognizing the theoretical construct between supervisor, peer, and self-rater MT scores, as well as observation of what MT is to a FF and how that affects occupation performance, specifically PAT performance.

Considering everything that was stated so far in relation to AF and/or MT components to predict PAT, Michaelides et al. stated that “fitness parameters used alone are not sufficient to fully predict a FF’s job performance” as there is 40% unexplained variation [32]. Examining further this 40% unexplained variation, it can be insinuated (a) that physiological parameters used alone cannot fully grasp the scope of a FF’s occupational demands and (b) that there are failings in holistically predicting occupational performance with absence of an integrated psychological measurement [32]. Therefore, the integration of both physiological and psychological parameters amongst the FF population was warranted and worth exploring to try and explain the remaining variation, as well as potentially providing a more interdisciplinary predictor of FF performance in the PAT. We believed that including multiple assessments capable of fully characterizing anaerobic performance (highlighting specific AF parameters, such as anaerobic power, peak anaerobic power, anaerobic capacity, and fatigue index) and incorporating a MT psychological component would better predict FFs’ performance in the PAT compared to a model including only AF parameters or only MT psychological components. Our research findings revealed that, despite an inability to implement a psychological parameter into the prediction model, a significant proportion (78%) of the variation observed in the PAT was explained by the variation of the AF parameters.

### 4.7. Current Physical Ability Test Modeling in Field and Laboratory Settings

Michaelides et al. [32] demonstrated that a five-variable subset model, which only included one AF parameter (measured via a 1 min anaerobic step test), contributed significantly to the predictive power of a FF’s PAT performance with a significant proportion (60%) of the variation observed in the PAT explained by the variation of the fitness parameters. Davis et al. [23] presented two models that involved field and laboratory fitness variables. Their research findings revealed better models for the evaluation of FFs’ general physical work capacity (based on five sequential firefighting tasks) when both laboratory and field tests were included (*R*^2^ = 0.90), compared to using field tests only (*R*^2^ = 0.54). However, aside from the research study being outdated, laboratory tests are complicated and expensive.

The demands of FFs are much different in the 21st century than they were 30 plus years ago. The need to revisit physiological characterization amongst FFs, coupled with varying resources regarding the physical testing of FFs, particularly when using laboratory tests in different geographical locations and FF departments, makes the case for using field assessments. Field tests demonstrate that implementation allows for replicable testing and cohort-sized applicability. They are also occupationally-specific, as well as time-efficient and economically feasible amongst tactical athletes, such as FF populations [25]. Lindberg et al. [62] concluded that field and laboratory tests could equally predict physical work capacities for firefighting work tasks, and models excluding anthropometric data were valid. William-Bell et al. [40] found that, aside from absolute VO_2max_, HG strength measured via a HG dynamometer was the next significant predictor of FF performance in CPAT (*R*^2^ = 0.47, *p* < 0.001), and they agreed with Davis and Dotson [23], who incorporated HG strength into both their prediction models. The William-Bell et al. [40] model was capable of explaining 65 to 71% of the variance in CPAT completion time amongst candidate FFs, respectively. The authors did not report a relationship between anaerobic capacity and CPAT performance; however, no differences were found in anaerobic fatigue resistance between individuals who completed CPAT and those unable to complete CPAT [40]. These research findings are contradictory to that of Sheaff et al. [39], who found that anaerobic fatigue resistance and absolute VO_2max_ combined best predicted CPAT performance (*R*^2^_adj_ = 0.82, *p* < 0.001), explaining a novel total variance in CPAT performance of 82% [39]. The discrepancy between the research findings of Sheaff et al. [39] and those of Williams-Bell et al. [40] may be explained by differences in research methodology and purpose. Sheaff et al. [39] utilized actual FFs to focus on the extent to which their physiological attributes predicted their CPAT performance, whereas Williams-Bell et al. [40] used volunteers with no prior firefighting experience to focus on the physiological demands of the CPAT. The experimental procedure of the Sheaff et al. [39] research study was intended to minimize the influence of skill acquisition as a potential confounding variable by using FFs familiar with all the tasks comprised in the CPAT. Sheaff et al. [39] also controlled for fatigue effects, similarly to our research study, by separating tests that could impair performance on subsequent tests. Williams-Bell et al. [40] assessed VO_2max_, muscular strength, and endurance testing, all of which preceded the Wingate test during the single day testing battery and which could have influenced Wingate performance and the relationship between Wingate performance and CPAT performance. Regardless of research findings, all the studies mentioned above did not meet test selection occupational specificity with assessments that, for the most part, were laboratory-based and did not acknowledge/incorporate performance testing in full occupational FF gear, which imposes considerable physiological burden due to its weight, insulative properties, and restrictiveness. The prior research studies also used singular AF assessments and, therefore, did not completely capture anaerobic performance, specifically with regards to assessing the different AF parameters (i.e., anaerobic power, peak anaerobic power, anaerobic capacity, and fatigue index) [39,93]. Even considering an adjusted *R*^2^ value, our research study’s prediction model was still capable of producing a significant proportion (75%*; p* < 0.001) of the variation observed in the PAT all while incorporating a battery of AF assessments that capture the full anaerobic capabilities of our participants.

Based on the models of Williams-Bell et al. [40], Sheaff et al. [39], and Michaelides et al. [32], approximately 30 to 40% of variance remains unexplained. We believe that our research study design achieved high specificity capable of meeting the demands of the occupation (i.e., test selection relevant and specific to population, incorporation of full occupational firefighting gear, and the use of multiple assessments to capture the different dimensions of AF). Despite the PAT performance showing significant correlation with all the AF parameters within our research study’s results, the predictive regression equation included only two variables: the maximal VJ, represented in absolute peak power, and the 300-yard shuttle run test time completion. Modeling a relationship between two variables is easy, but not practical, as firefighting is complex in nature. Statistically speaking, a prediction model may have more power when more significant predictors are included [56,57,94]. However, our research study’s model was capable of explaining a novel 78% (*R^2^*) of the variation observed in the PAT performance, which was explained by the variation of these two AF parameters only (i.e., VJ and 300-yard shuttle run).

The ability of a maximal VJ to assess different AF parameters (i.e., specifically anaerobic power and peak anaerobic power), particularly with relation to the task performance requiring lower body explosiveness and power, is essential to FFs. The inclusion of the maximal vertical jump in predictive regression equations is confirmed by Hauschild et al. [25] also found that jump tests are shown to be commonly entered into predictive regression equations in the research studies reviewed [25,59,77]. More specifically, Harman et al. [77] found VJ (aside from anthropometric measures) to be consistently entered within several predictive regression equations of simulated battlefield physical performance from field-expedient tests amongst 32 civilian males. More recently, the VJ test was included in the newest Canadian military basic fitness test battery, demonstrating its potential feasibility for AF assessment and parameters [95].

The 300-yard shuttle run test, similar to the 400-m run used by Rhea et al. [66], lasts between 40 to 90 s in assessment duration [48,49]. A FF must not only demonstrate a high aerobic capacity, but also anaerobic resistance to fatigue, so that, not only may the FF work anaerobically at high levels, but it can also regenerate quickly and perform anaerobically again. A large absolute VO_2max_, as confirmed by Williams-Bell et al. [40], may allow the FFs to meet occupational energy demands without significant activation of anaerobic metabolic pathways, thereby preventing or delaying fatigue. However, because many firefighting tasks, such as forcible entry or ladder retrieval, require a sustained high force and metabolic output, it is logical that the 300-yard shuttle run (specifically, highlighting AF parameters—anaerobic power, peak anaerobic power, anaerobic capacity, and fatigue index) would be indicative of a FF’s PAT performance. FFs with greater AF may be able to continue to function at a high level during the firefighting emergency scenarios, while less anaerobically fit individuals may be required to cease current tasks to recover from prior maximal and anaerobic exertion.

### 4.8. Limitations

There were several limitations in the current research study starting with the variance in departmental PATs used (i.e., CGFD and HFD). The number and type of task performed in the PAT were defined as most relevant occupation-related tasks by the specific department. Non-AF parameters known to contribute to a FF’s performance were not assessed (i.e., aerobic and flexibility parameters). Only one psychological component, specifically, MT was assessed. Subsequently, there is no MT measurement instrument specifically designed for the FF population. The research study also did not control for previous firefighting experience (i.e., rank differences), discrepancies concerning fitness assessments with relation to age differences, previous fitness levels, nor dietary habits throughout the entire duration of the research study. FFs were eligible to participate if they were non-prospective/candidate FFs (i.e., fire academy participants), active fire suppression personnel, and between the ages of 20 to 55 years. No female FF participants were included in the research study, thereby limiting our ability to make accurate and definitive determinations of sex differences in our results. All the prior limitations coupled with a global COVID-19 pandemic, a period where the data collection process was completed, and which resulted in a relatively small sample size of randomly selected FFs from different departments, which may have limited the scope of the population for which the results can be generalized. Therefore, our current research study may be underpowered based on our limited sample size and based on deeming the research exploratory in nature. Lastly, due to the cross-sectional design of the current research study, we are unable to determine causal or independent relationships between specific physical attributes and PAT performance.

A statistically more powerful research study with a larger sample size may increase the likelihood to detect the effect of the evaluated independent variables with a FF’s PAT scores, assuming there is an effect. In turn, this may allow a better understanding of the anaerobic performance along with the input of MT levels and their relationship to firefighting performance as evaluated by the PAT. Clarification of such parameters may then lead to the development of a firefighting assessment, including both AF parameters and MT psychological component instruments, which can be implemented and conducted by fire training facilities/departments. This will enable FFs and instructors to prepare more adequately for both the physical and mental aspects of their job. Understanding the physiological and psychological parameters that produce optimal performance, as evaluated by the PAT, will: provide a more effective and accurate measure to evaluate performance in FFs; enable FFs to concentrate physical strength and conditioning efforts on those specific variables that predict high performance; add to the growing body of research supporting FF safety and mental health; and contribute to the continued development of physical fitness recommendations and mental health guidelines amongst FFs.

### 4.9. Directions for Futured Research

Future investigations should also research cohorts of female gender FFs to confirm whether the physiological attributes in this current research study tend to influence PAT performance time scores differently than their male gender counterpart. Resilience should be considered as an alternative to MT [96]. Future research should also seek to establish independent effects by using interventions, such as exercise training programs and control groups, to isolate changes in independent physiological attributes and to control for other intervening factors that could influence PAT performance time scores. Lastly, future investigation should explore and characterize FFs’ psychological profiles, as well as develop specific psychological component assessment scales for FFs.

## 5. Conclusions

Our research findings demonstrated that anaerobic fitness parameters were shown to be significantly correlated with PAT performance. The handgrip dynamometry, maximal vertical jump, and the Margaria-Kalamen staircase test all demonstrated negative correlation. The 300-yard (274 m) shuttle run test demonstrated a positive correlation. Despite an inability to implement a psychological parameter into the prediction model, only the anaerobic fitness parameters (78%) were used as determinants in the PAT prediction model. It is imperative that firefighters optimize their anaerobic performance (highlighting specific anaerobic fitness parameters, such as anaerobic power, peak anaerobic power, anaerobic capacity, and fatigue index), which will in turn allow for enhanced occupational performance and readiness as evaluated by the PAT. Further research is needed in respect to MT psychological concept in firefighters and its relationship with their occupational performance.

## Figures and Tables

**Table 1:** Descriptive Statistics—Anthropometrics & Body Composition.

	Mean ± SD	Min	Max
Age (years)	29.0 ± 7.0	20.0	45.0
Height (m)	1.7 ± 0.1	1.6	1.8
Weight (kg)	79.9 ± 13.0	61.2	108.9
Occupational Weight (kg)	102.6 ± 13.0	83.9	131.5
BMI (kg/m^2^)	26.3 ± 2.7	21.8	33.6
BF (%)	17.9 ± 5.6	8.0	28.7
WHR	0.9 ± 0.1	0.8	1.3

Min: minimum; Max: maximum; SD: standard deviation; Weight: represents participant in shorts, t-shirt, and a pair of comfortable jogging/running shoes; Occupational Weight: represents participants weight with full occupational FF gear; BMI: body mass index; BF: body fat percentage; WHR: waist-to-hip ratio represents the circumference of the waist divided by the circumference of the hips.

**Table 2:** Descriptive Statistics—PAT Scores and AF Parameters.

	Mean ± SD	Min	Max
PAT (mm:ss)	04:07 ± 01:22	02:09	06:34
HG (kg)	33.1 ± 7.8	20.0	50.0
VJAPP (W)	5703.6 ± 913.0	4469.3	7870.6
MKAP (W)	794.5 ± 232.7	344.0	1281.2
300YD (mm:ss)	01:21 ± 00:06	01:13	01:39

Min: minimum; Max: maximum; SD: standard deviation; PAT: physical ability test; HG: handgrip dynamometer absolute strength; VJAPP: vertical jump absolute peak power; MKAP: Margaria-Kalamen staircase test absolute power; 300YD: 300-yard (274 m) shuttle run test.

**Table 3:** Descriptive Statistics—MT Psychological Component Instruments and MT Levels.

	Mean ± SD	Min	Max
SMTQ	46.3 ± 4.0	38.0	52.0
MTMTI	15.7 ± 2.4	12.0	20.3

Min: minimum; Max: maximum; SD: standard deviation; SMTQ: sports mental toughness questionnaire; MTMTI: military training mental toughness inventory.

**Table 4:** Pearson’s correlations between PAT Scores, AF Parameters, and MT Levels.

	PAT (mm:ss)	HG (kg)	VJAPP (W)	MKAP (W)	300YD (mm:ss)	SMTQ	MTMTI
PAT (mm:ss)	1.000	−0.708 **	−0.734 **	−0.752 *	0.599 *	−0.330	0.212
HG (kg)	−0.708 **	1.000	0.690 **	0.649 *	−0.554 *	−0.115	−0.060
VJAPP (W)	−0.734 **	0.690 **	1.000	0.781 *	−0.156	0.224	−0.181
MKAP (W)	−0.752 **	0.649 *	0.781 **	1.000	−0.420	0.314	−0.297
300YD (mm:ss)	0.599 *	−0.554 *	−0.156	−0.420	1.000	0.000	0.036
SMTQ	−0.330	−0.115	0.224	0.314	0.000	1.000	−0.677 *
MTMTI	0.212	−0.060	−0.181	−0.297	0.036	−0.677 *	1.000

** Sig. (2-tailed) *p* < 0.01; * Sig. (2-tailed) *p* < 0.05; PAT: physical ability test; HG: handgrip dynamometer absolute strength; VJAPP: vertical jump absolute peak power; MKAP: Margaria-Kalamen staircase test absolute power; 300YD: 300-yard (274 m) shuttle run test; SMTQ: sports mental toughness questionnaire; MTMTI: military training mental toughness inventory.

**Table 5:** Model Summary—Predicting PAT Scores from AF Parameters and/or MT Levels.

Model	R	R²	Adj. R²	SEE	Change Statistics					Durbin-Watson
					R² Change	F Change	df1	df2	Sig. F Change	
1	0.887	0.786	0.747	41.685	−0.035	1.965	1	10	0.191	2.386
Predictors: (Constant), 300YD, VJAPP										
Dependent Variable: PAT										

Adj: adjusted; SEE: standard error of the estimate; PAT: physical ability test; HG: handgrip dynamometer absolute strength; VJAPP: vertical jump absolute peak power; MKAP: Margaria-Kalamen staircase test absolute power; 300YD: 300-yard (274 m) shuttle run test; SMTQ: sports mental toughness questionnaire; MTMTI: military training mental toughness inventory.

**Table 6:** Coefficients—PAT Scores, AF Parameters and MT Levels.

Model		Unstand. Coeff.		Stand. Coeff.	t	Sig.	95.0% CI for B [Lower, Upper Bound]	Correlations			Collinearity Statistics	
		B	SE	Beta				Zero-Order	Partial	Part	Tolerance	VIF
1	(Constant)	30.078	169.638		0.177	0.862	[−343.293, 403.449]					
	VJAPP	−0.060	0.013	−0.657	−4.691	0.001	[−0.088, −0.032]	−0.709	−0.817	−0.654	0.991	1.009
	300YD	6.894	1.804	0.535	3.821	0.003	[2.922, 10.865]	0.599	0.755	0.533	0.991	1.009
Dependent Variable: PAT												

CI: confidence interval; Coeff: coefficients; SE: standard error; Unstand: unstandardized; stand: standardized; PAT: physical ability test; HG: handgrip dynamometer absolute strength; VJAPP: vertical jump absolute peak power; MKAP: Margaria-Kalamen staircase test absolute power; 300YD: 300-yard (274 m) shuttle run test; SMTQ: sports mental toughness questionnaire; MTMTI: military training mental toughness inventory.

## Data Availability

Data are available on request due to restrictions, e.g., privacy or ethical concerns.

## Supplementary Materials

The following supporting information can be downloaded at: https://www.mdpi.com/article/10.3390/ijerph192215253/s1. S1.1.1. CGFD PAT; S1.1.2. HFD PAT; Table S1: ANOVA–PAT Scores, AF Parameters and MT Levels.
