# Supplementary material for: Predicting Firefighters’ Physical Ability Test Scores from Anaerobic Fitness Parameters & Mental Toughness Levels
Source: Int J Environ Res Public Health. 2022 Nov 18;19(22):15253. doi: 10.3390/ijerph192215253 (PMC9691205; doi:10.3390/ijerph192215253)
Supplement: Supplementary file 1 [file ijerph-19-15253-s001.zip › ijerph-1999009-supplementary.pdf]

SUPPLEMENTARY MATERIAL

S1. Procedures

S1.1. Experimental Condition 1 – Physical Ability Test (PAT)

S1.1.1. Coral Gables Fire Department (CGFD) PAT

The consecutive CGFD PAT tasks are described as follows:

Task 1 – Stair Climb

This event was designed to simulate the critical tasks of climbing stairs while carrying a high-rise hose in full protective gear carrying firefighter equipment. Firefighters climbed (i.e., making contact with every step) on a Stair Master Step Mill® at a set stepping rate of 60 steps per minute (Level 4) for 3 minutes. Prior to the initiation of the timed PAT, the firefighter had a 20 second warm-up on the Stair Master Step Mill® at a set stepping rate of 50 steps per minute (Level 3). During this warm-up period, the firefighter was permitted to dismount, grasp the rail, or hold the wall to establish balance and cadence. If the firefighter fell or stepped off the Stair Master Step Mill® during the 20 second warm-up period, the firefighter was required to remount the Stair Master Step Mill® and restart the entire 20 second warm-up period. The firefighter was allowed to restart the warm-up period twice. There was no break in time between the warm-up period and the actual timing of the test. The timing of the test began at the end of the warm-up period when the test administrators/evaluators called out "START." After the 3 minutes, the task was concluded. The firefighter walked 25.91 m (85 ft) within the established walkway to the next task.

Task 2 – Hose Drag

This event was designed to simulate the critical tasks of dragging an uncharged hose line from the fire apparatus to the fire occupancy and pulling an uncharged hose line around obstacles while remaining stationary. The firefighter grasped an automatic nozzle attached to 60 m (200 ft) of 44 mm (1 3/4-inch) hose. The firefighter placed the hose line over the shoulder or across the chest, not exceeding the 2.44 m (8 ft) mark. Hands were placed on the hose or coupling, however not the nozzle. The firefighter was permitted to run during the hose drag. If a firefighter were to have slipped, they were allowed to get up and continue the task. The firefighter dragged the hose 22.86 m (75 ft) to a prepositioned drum, made a 90° turn around the drum and continued an additional 7.62 m (25 ft). The firefighter then stopped within the marked 1.52 m × 2.13 m (5 ft ×

7 ft) box, dropped to at least one knee, and pulled the hose line until the hose line's 15.24 m (50 ft) mark was across the finish line. During the hose pull, the firefighter must have kept at least one knee in contact with the ground and knee(s) must remain within the marked boundary lines. The evolution (task) was finished when the nozzle completely crossed the line between the cones and placed on the ground. The firefighter walked 25.91 m (85 ft) within the established walkway to the next task.

Task 3 – Equipment Carry

This event was designed to simulate the critical tasks of moving heavy equipment tools, carrying them to the emergency scene and returning them. The firefighter removed the two saws from the tool cabinet, one at a time, and placed them on the ground. The firefighter then picked up both saws, one in each hand, and carried them while walking 22.86 m (75 ft) around the drum, then back to the starting point. The firefighter was permitted to place the saw(s) on the ground and adjust the grip. Upon return to the tool cabinet, the firefighter placed both saws on the ground, then picked up each saw one at a time, and replaced the saw in the designated space in the cabinet. Completion of the task required proper carrying technique and control for the duration of carrying and placement of the saws by the firefighter at all times. The firefighter walked 25.91 m (85 ft) within the established walkway to the next task.

Task 4 –Ladder Raise And Extension

This event was designed to simulate the critical tasks of placing a ground ladder at a fire structure and extending the ladder to the roof or window. The firefighter walked to the top rung of the 7.32 m (24 ft) aluminum extension ladder, which was footed by retractable safety lanyard, lifted the first rung at the unhinged end from the ground, and walked it up until it was stationary against the wall. This was done in a hand over hand fashion, using each rung until the ladder was stationary against the wall. The firefighter was not to use the ladder rails to raise the ladder. The firefighter immediately proceeded to the pre-positioned and secured 7.32 m (24 ft) aluminum extension ladder, stood with both feet within the marked box of 91.44 cm × 91.44 cm (36 in × 36 in) and extended the fly section hand over hand until it hit the stop. The firefighter then lowered the fly section hand over hand in a controlled fashion to the starting position. This concluded the

task. Proper technique was to be employed for the duration of the task. The firefighter walked 25.91 m (85 ft) within the established walkway to the next task.

Task 5 - Forcible Entry Evolution

This event was designed to simulate the critical tasks of using force to open a locked door or to breach a wall. During this event, the firefighter used a 4.54 kg (10 lb) sledgehammer and struck the measuring device in the target area until the buzzer signal was activated. The firefighters' feet were to remain outside the toe-box. After the buzzer was activated, the firefighter placed the sledgehammer on the ground. This concluded the task. If at any time a participating firefighter lost control of the sledgehammer or threw the hammer on the ground, the result was immediate disqualification. The firefighter walked 25.91 m (85 ft) within the established walkway to the next task.

Task 6 - Search

This event was designed to simulate the critical tasks of searching for a fire victim with limited visibility in an unpredictable area. Firefighters crawled (i.e., on their hands and knees) through a darkened tunnel maze with dimensions approximately 91.44 cm (3 ft) high, 121.92 cm (4 ft) wide and 19.51 m (64 ft) in length including both unobstructed and obstructed (i.e., obstacles) portions. Firefighters' movement/behavior were monitored for arousal regulation and control. Throughout the tunnel maze, firefighters were guided and adhered to the verbal instructions given by the test administrators/evaluators to navigate around, over, and under obstacles. In addition, at two locations, firefighters crawled through narrowed spaces where the dimensions of the tunnel were reduced. The task began when firefighters' hands/knees were placed on the ground while preparing to enter the maze. The task was completed upon the firefighters return and exit from the maze. The firefighter walked 25.91 m (85 ft) within the established walkway to the next task.

Task 7 - Rescue

This event was designed to simulate the critical tasks of removing a victim or injured partner from a fire scene. The firefighter grasped a 74.84 kg (165 lb) mannequin by the handle(s) on the shoulder(s) of the harness (either one or both handles were permitted), dragged it 10.67 m

(35 ft) to a pre-positioned drum, made a 180° turn around the drum, and continued an additional 10.67 m (35 ft) to the finish line. If a firefighter should have slipped during the task, they were allowed to get up and continue. The firefighter was not permitted to grasp or rest on the drum. It was permissible for the mannequin to touch the drum. The firefighter was permitted to lower the mannequin to the ground to adjust their grip. The entire mannequin must be dragged past the marked finish line. This concluded the task. The firefighter walked 25.91 m (85 ft) within the established walkway to the next task.

Task 8 – Ceiling Breach and Pull

This event was designed to simulate the critical tasks of breaching and pulling down a ceiling to check for fire extension at a fire scene. Firefighters removed a 1.83 m (6 ft) pike pole from the bracket, stood within the boundary established by the equipment frame, and placed the tip of the pole on the painted area of the hinged door in the ceiling. The firefighter then fully pushed up a 27.22 kg (60 lb) hinged door in the ceiling with the pike pole three times. The firefighter then hooked the pike pole to the 36.29 kg (80 lb) ceiling device and pulled the pole down five times. Each set consisted of three pushes and five pulls. The firefighter repeated the set four times. The firefighter was permitted to stop and, if needed, adjust the grip. Releasing the grip or slipping from pike pole handle, without the pike pole falling to ground, did not result in a warning nor constituted as a failure. The firefighter was allowed to re-establish the grip and resume the event. If the firefighter did not successfully complete a repetition (i.e., complete the up and down motion), the test administrators/evaluators called out "MISS" and the firefighter had to push or pull the apparatus again to complete the repetition. The task and the total PAT time ended when the firefighter completed the final pull stroke repetition as indicated by the test administrators/evaluators who called out "TIME".

S1.1.2. Hialeah Fire Department (HFD) PAT

The consecutive HFD PAT tasks are described as follows:

Part One

Task 1 - High-Rise Stair-Climb Evolution

The firefighter started at the base of the training tower. The high-rise hose pack was placed on the shoulder with the hand from that shoulder placed on top to secure the high-rise

hose pack. The freehand was to be used on the handrail for balance only. The left shoulder was used to carry the hose pack on the way up the stairs, and the right shoulder was used on the way down. The handrail was not to be used by the firefighter to pull themselves up or pivot around the landings. Contact was made with every step. The firefighter was not allowed to skip a step ascending or descending. The hose pack was deposited on the 5th-floor landing on the designated spot as instructed by the test administrators/evaluators. This concluded the task.

Task 2 - Hoist Evolution

The firefighter walked to the 5th-floor balcony and stepped out onto the balcony. Standing behind the line, they used a hand-over-hand method to pull the rolled hose to the top of the railing. They then walked forward, grabbed the hose roll, and lifted the hose roll over the railing placing it on the floor. Firefighters were not allowed to place their foot on the railing to assist in the hoist. The firefighter then returned to the 5th-floor landing and placed the high-rise hose pack on the shoulder. The firefighter descended the stairs using the handrail for balance only. Contact was made with every step, not skipping a step, or running at any time. Once at the base of the stairs, the firefighter placed the high-rise hose pack on the ground and proceeded to the Forcible Entry Evolution. Control of the high-rise pack was to be maintained at all times. If at any time the high-rise pack dropped from the firefighters' shoulder or the firefighter threw the high-rise pack down to the ground, result was immediate disqualification.

Task 3 - Forcible Entry Evolution

The firefighter positioned both feet on the Kaiser Sled so that their toes were even with the weighted steel slide block. The firefighter bent forward so that they could see the end of the slide at all times. With the 3.6 kg (8 lb) dead-blow hammer, using short hard strokes, the firefighter drove the sled 1.524 m (5 ft) until it cleared the markings. The test administrators/evaluators advised the firefighter when the task was completed. The body was to be kept in the bent forward position and the eyes on the target at all times. The hammerhead could not be raised higher than the level of the head. Once the task was completed, the firefighter carefully set the hammer down and proceeded to the Hose Advance Evolution. The firefighter walked 25.91 m (85 ft) within the established walkway to the next task. If at any time a

participating firefighter lost control of the hammer or threw the hammer onto the ground, the result was immediate disqualification.

Task 4 - Hose Advance Evolution

The firefighter picked up the nozzle and placed it over either shoulder. They dragged the charged 4.445 cm (1.75 in) hose line straight forward, 22.86 m (75 ft). The evolution was finished when the nozzle completely crossed the line between the cones. If a firefighter should have slipped, they were allowed to get up and continue. Hands were placed on the hose or coupling, not the nozzle, and the nozzle should reach the waist. Automatic failure occurred if the nozzle was dropped or if the firefighter was unable to complete the hose advance evolution. Once completed, the firefighter placed the nozzle on the ground and walk around the cones to the Victim Rescue Evolution. The firefighter walked 25.91 m (85 ft) within the established walkway to the next task.

Task 5 - Victim Rescue Evolution

The firefighter picked up a 79.37 kg (175 lb) mannequin and dragged it, walking the victim, i.e., the mannequin backward 30.48 m (100 ft) to the marked finish line. This evolution was completed when the victim completely cleared the finish line. If firefighter should have slipped, they are allowed to get up and continue. Upon completion of the victim rescue evolution (task 5), the firefighter will have completed part one. The firefighter walked 25.91 m (85 ft) within the established walkway to the next task. Part two had no time limit and began with task six.

Part Two

Task 6 - Ladder- One Person Low Shoulder Carry

Standing behind the cone, the firefighter picked up a 7.315 m (24 ft) extension ladder and used a low shoulder carry to walk a distance of 15.24 m (50 ft) to a marked area. The firefighter was to maintain control of the ladder at all times. The task started when the firefighter positioned themselves at lifting point near the center of the ladder. The firefighter kneeled beside the ladder at the lifting point facing the heel of the ladder on knee closest to the ladder. The firefighter grasped the ladder at or near the middle rung opposite to their knee with the hand closest to the

ladder, with palm forward and stood the ladder on the edge. The firefighter pivoted ladder onto the nearer beam, raising the farther beam. The firefighter stood up and picked up, using leg muscles, keeping their back straight and vertical. As the ladder was brought up, firefighter pivoted toward butt end of the ladder and inserted their other arm through rungs. The firefighter positioned ladder for carrying with the upper beam resting on their shoulder. The firefighter lowered butt end slightly and steadied the ladder with both their hands. The firefighter carried the ladder to the designated area and placed the ladder on the ground (control of the ladder was to be maintained at all times). The firefighter then reversed the lifting procedure. The firefighter lowered the ladder to the ground. This concluded the task. The firefighter walked 25.91 m (85 ft) within the established walkway to the next task.

Task 7 - 35' Ladder Climb with Leg Lock

The firefighter walked to a 10.668 m (35 ft) extension ladder which was footed by safety lines. The firefighter climbed to the desired height as instructed by the test administrators/evaluators, put in a leg lock with the leg indicated by the test administrators/evaluators, and touched the red marker with their right hand. The firefighter then descends the ladder. Safety lines were removed once firefighter was safely on the ground. Proper technique was to be employed for the carrying and placement of the 7.315 m (24 ft) ladder and the climbing of the 10.668 m (35 ft) ladder. This concluded the task. The firefighter walked 25.91 m (85 ft) within the established walkway to the next task.

Task 8 - Extrication Exercise

Using the appropriate handles, the firefighter picked up the Genesis Spreader from the simulated truck tray. They then walked to the vehicle and touched the tips to the designated areas as instructed by the test administrators/evaluators (four points of contact on each side of the vehicle). The firefighter was to keep the tips perpendicular to the vehicle at all times. Once the firefighter had completed all designated tasks with the spreaders, they returned the tool to the point of origin. This concluded the task. The firefighter walked 25.91 m (85 ft) within the established walkway to the next task.

Task 9 - Confined Space Crawl Exercise

The firefighter crawled through an unobstructed portion of a darkened maze. Within the maze are obstacles. The task began when the firefighter placed a hand or knee on the ground while preparing to enter the maze and adhered to the instructions given by the test administrators/evaluators. During this event, the firefighter crawled on their hands and knees through a tunnel maze that is approximately 91.44 cm (3 ft) high, 121.92 cm (4 ft) wide and 19.51 m (64 ft) in length with two 90° turns. At several locations in the tunnel, the firefighter navigated around, over, and under obstacles. Also, at two locations, the firefighter crawled through a narrowed space where the dimensions of the tunnel were reduced. The firefighters' movement were monitored/listened to as they advanced through the maze. The task and the PAT event were completed upon returning and exiting 60.96 cm (2 ft) from the maze.

S1.2. Experimental Condition 2 - Anaerobic Fitness (AF) Assessments

Handgrip (HG) Dynamometry

Procedural Steps for HG dynamometry are summarized as follows:

1. The firefighter was in the standing position.
2. The firefighters' head was in the mid-position (facing straight ahead).
3. The firefighters' forearm was placed at any angle between 90° and 180° (right angle to straight) of the upper arm; the upper arm hung in a vertical position.
4. The firefighters' wrist and forearm was at the mid prone position.
5. The firefighter exerted maximally and quickly after hearing the technician's following instructions:
    a. "Are you ready?"
    b. "Squeeze as hard as you can." As the firefighter begins,
    c. "Harder!... Harder!... Relax."
6. The firefighter performed three trials alternately with each hand, with at least 30 seconds or up to one minute between trials for the same hand.
7. The technician recorded the force in kg.
8. The technician reset the dynamometer's pointer to zero after each trail.
9. Calculations were performed to derive absolute maximum strength (kg) from best handgrip trial score.

Maximal Vertical Jump (VJ)

Procedural Steps for maximal VJ are summarized as follows:

1. The firefighter executed several practice jumps as part of the familiarization with the equipment.
2. The technician explained and demonstrated the proper position for the standing reach (it could be done with the dominant hand alone, or it could be done with the two hands held together).
    a. Standing with feet together and dominant side near the Vertec® equipment
    b. Reaching as high as possible with the dominant hand but keeping both feet flat on the floor
    c. Placing the palm of the hand against the Vertec® equipment (and pushing the vanes forward slightly)
3. The technician recorded the standing reach height (to the closest 0.5 in).
4. The technician explained and demonstrated the VJ and reached with a countermovement but no step.
    a. The firefighter lined up within 2 m (6.56 ft) from the Vertec® equipment.
    b. The firefighter initiated the jump via a dip down into a semi-crouching position (the countermovement), then jumped maximally off both feet straight up from under the Vertec® equipment while simultaneously swinging both arms forcefully upward.
    c. It was essential to coordinate the jump so that the firefighter contacted the Vertec® equipment at the peak height of the jump.
    d. The firefighter was to land with the knees bent to help safely absorb the landing forces.
5. The firefighter completed at least three maximal trials (or continued until the jumping reach height plateaus) with 60 seconds rest between trials.
6. The technician recorded each trial or simply the highest jumping reach height (to the closest 0.5 in) and later converted to metric unit.
7. Calculations were performed to derive jump distance (difference) and absolute peak power.

Margaria-Kalamen (MK) Staircase Test

Procedural Steps for MK staircase test are summarized as follows:

1. The technician placed cones on steps three and nine and put a mark on the floor six meters from the bottom of the first step. Technicians used a handheld stopwatch at the designated steps to determine the firefighters' time.
2. The firefighter performed several practice-runs up the stairs to learn how to step on every third step.
3. The technician initiated the first test three minutes after completing the last practice run.
4. The technician instructed the firefighter to run up the stairs as quickly as possible.
5. The technician started when the firefighter stepped on step three and stops when the firefighter stepped on step nine. Due to using a handheld stopwatch, the technician started the watch when the firefighter stepped on step three and stopped it when the firefighter crossed step nine.
6. The technician recorded the time.
7. The test was repeated three minutes after completing the first trial. The second time value of the firefighter was recorded.
8. The mean time was calculated based on trial one and trial two. The result was recorded.

Calculations were performed to derive mean time, absolute power for each individual trial, and mean time absolute power from the average of the two trial scores.

300-yard (274 m) Shuttle Run

Procedural Steps for the 300-yard shuttle run are summarized as follows:

1. The firefighters were paired off by similar ability.
2. The firefighters were positioned immediately behind one line, facing the other line.
3. On an auditory signal, the firefighters' sprinted to a line 25 yd (22.86 m) away, making foot contact with it, then immediately sprinted back to the first line. Six such round trips were made as fast as possible without stopping (6 × 50 yd = 300 yds, or 274 m).
4. On completion of the first trial, both firefighters' times were recorded to the nearest 0.1 seconds via a handheld stopwatch, and a five-minute rest interval was initiated using the same handheld stopwatch. As each pair of firefighters completed the first trial, they were allowed to walk and stretch but were to stay alert for the starting time on the second trial.

5. The average of two trials were recorded to the nearest 0.1 seconds.

6. Calculations were performed to derive mean time.

S2. Results

Table S1. ANOVA– PAT Scores, AF Parameters and MT Levels

Model		Sum of Squares	df	Mean Square	F	Sig.
1	Regression	70265.519	2	35132.759	20.218	.000[f]
	Residual	19114.196	11	1737.654		
	Total	89379.714	13			

Predictors: (Constant), 300YD, VJAPP
Dependent Variable: PAT

PAT: Physical ability test; HG: Handgrip dynamometer absolute strength; VJAPP: Vertical jump absolute peak power; MKAP: Margaria-Kalamen staircase test absolute power; 300YD: 300-yard (274 m) shuttle run test; SMTQ: Sports mental toughness questionnaire; MTMTI: Military training mental toughness inventory.
